# Evaluating the Efficacy of *Achillea millefolium *and *Thymus vulgaris* Extracts Against Newcastle Disease Virus *in Ovo*

**DOI:** 10.5812/jjm.9016

**Published:** 2014-02-01

**Authors:** Seyedeh Elham Rezatofighi, Akram Seydabadi, Seyyed Mansour Seyyed Nejad

**Affiliations:** 1 Department of Biology, Faculty of Science, Shahid Chamran University of Ahvaz, Ahvaz, IR Iran

**Keywords:** *Achillea millefolium*, *Thymus vulgaris*, Newcastle Disease Virus, Antiviral Activity

## Abstract

**Background:**

Nowadays natural products such as pure compounds and plant extract scan provide unlimited opportunities for new antiviral drugs. Newcastle disease virus (NDV) is one of the most important viral diseases in poultry industry. Vaccination could provide protection against NDV outbreaks, but it is not sufficient because infections by NDVs have remained frequent around the world.

**Objectives:**

The current research aimed to study *Achillea millefolium* and *Thymus vulgaris* antiviral activity against Newcastle disease virus (NDV).

**Materials and Methods:**

The antiviral activity of the plants was measured by the reduction assay of viral titer, and explained by inhibition percentage (IP).

**Results:**

Inhibition percentage was determined as 10 ^1.75^, which indicated the ability of the extracts to reduce the viral potency by more than 56 folds.

**Conclusions:**

Both plants were found effective against Newcastle disease virus.

## 1. Background 

Pseudo-fowl pest or Newcastle disease (ND), a devastating disease of poultry seen in chickens and turkeys, caused by Newcastle disease virus (NDV). The signs of disease are high mortality, hemorrhagic intestinal lesions, severe respiratory distress, decrease of egg production, and nervous disorders (1). The NDV injected in embryonated eggs could grow in cells lining the allantoic cavity. The virus grows in these cells, destructs them and is then released in the allantoic fluid reaching high titers in approximately 24 hours. If virulent NDV strains are inoculated, most of the embryos die two days after inoculation. Injected NDV causes remarkable histopathological changes in dead or alive embryos (1). 

Vaccination programs can provide protection against NDV outbreaks, but they are not sufficient because infections by NDVs have remained frequent around the world in the recent years (2). There is no known specific treatment for NDV, like other viral diseases (3). Several antiviral drugs are known to treat mammalian viruses, however their use in avian disease are limited because these agents may be toxic for the host cells (3). Although there is little documentation, there has been experimental evidence regarding the ability of several plants to treat numerous diseases (3). 

Nowadays, choosing plants and their materials is focused on their use in traditional or modern medicine (4). *Achillea millefolium* (yarrow) is a powerful medicinal plant which is widely distributed and has been used by different cultures for thousands of years (5). *A. millefolium* seems to originate from European folk medicine and had later spread to the east. According to the studies conducted on yarrow, it has anti-inflammatory, antitumor, antioxidant, antibacterial and antiviral activities (6). *Thymus vulgaris* (thyme), a Mediterranean aromatic plant, has been frequently used for medicinal purposes. *T. vulgaris* has been used in traditional medicine for several of its properties such as antiseptic, antimicrobial, antifungal, antioxidative, and antiviral activities (7).

## 2. Objectives 

The current study aimed to evaluate the efficacy of the *A. millefolium* and *T. vulgaris* extracts against NDV in embryonated eggs.

## 3. Materials and Methods

### 3.1. Plant Material and Extract Preparation

The plant samples were identified by a plant taxonomist at the Department of Biology, Shahid Chamran University of Ahvaz, Iran. The plants were shade dried at room temperature for 10 days. *A. millefolium* flowers and *T. vulgaris* leaves were used in this study since these parts are used in traditional medicine. The aforementioned parts were ground to a fine powder. One gram of powder was extracted using 10 mL of ethanol/distilled water solution (alcohol/water = 8:2, v/v), with centrifugation at 3000 r/min for 15 minutes, and then the supernatant was collected. This process was repeated three times. Solvents were then removed by evaporation (8, 9). Since the solvent composed of alcohol and water, it completely evaporated and no moisture remained in the next step of the study.

### 3.2. Extracts Biosafety

 For the safety of their future use as therapeutic agents, these extracts must not have any toxicity against the tested host. Therefore plant extracts were assayed for egg embryonated toxicity. To this aim, dilutions of 10, 50, 100, 200, 400, 800, and 1000 μg/mL of extracts were assayed and the maximum non-toxic concentration (MNTC) was used for *in vitro* antiviral screening test. Then, 100 μL of each concentration was injected to allantoic cavity of 7-dayembryonated eggs. Eggs were incubated at 37˚C for two weeks. If extracts were not toxic for eggs, the chickens would be born alive and healthy.

### 3.3. Antiviral Activity of Extracts

A field strain of NDV was obtained from the veterinary medicine department of the University of Tehran. This virus was isolated from fowl disease. Stock suspensions of the virus were prepared in the following manner. The received virus was inoculated to 9-day embryonated eggs. After 4 days the allantoic fluid was harvested and HA test was applied to confirm the virus (10).

The antiviral activity of plant extracts was assayed in the following manner. The stock allantoic fluid suspension of the virus was diluted in concentrations of 10-1, 10-2, 10-3, 10-4 and 10-5 in sterilized phosphate buffer saline. Next, 500μLof the plant extracts were mixed separately with 500μLof the diluted concentrations of virus and the mixture was incubated for one hour at 4°C to allow reaction occurrence (11). Then 200 μL of extract/virus was inoculated to allantoic cavity of 7-dayembryonated heneggs. All different concentrations of the virus were also injected to embryonated eggs. Uninoculated eggs served as negative controls whereas eggs with virus suspension but free of the extract served as positive controls. 

Triplicate tests were applied for each virus concentration with and without the extract (12). Next, the eggs were returned to the incubator. The allantoic fluid was harvested five days after inoculation and analyzed for virus titer by a standard haemagglutination (HA) test (13). This test is a fast and simple method for the detection of NDV. Death of embryos which died within 24 hours after infection was considered nonspecific. The embryo infection dose 50 (EID50), and values of NDV suspensions were determined by the Reed and Muench method (12). For haemagglutination (HA) test 50 μL of the allantoic fluid was mixed with 50 μL of 1% chicken red blood cell on HA plate. The HA plate was gently rocked and observed for visible haemagglutination, indicating viral activity (13). This was done for every egg and the observations were recorded. Antiviral activity was determined with reduction of virus titers using EID50 determinant.

## 4. Results 

Egg toxicity assay was performed using embryonated eggs to determine the maximum non-toxic concentration (MNTC). The obtained MNTCof *A. millefolium* and *T. vulgaris* were 10 and 50 μg/mL concentrations, respectively. At higher concentrations, chickens were dead, but at lower concentrations they were born alive and healthy. Therefore, for the antiviral assay of *A. millefolium* and *T. vulgaris*, 10 and 50 μg/mL concentrations were selected, respectively. The results of the overall *in ovo* screening are summarized in Tables 1 and 2. Plant extracts were tested *in ovo* against NDV in allantoic cavity and their gross antiviral activity was evaluated in terms of haemagglutination inhibition. 

The extracts were considered active if they reduced EID50 of the virus in the case of NDV, and EID50 of the virus and the virus/extract was measured by the Reed and Muench method. Antiviral activities were measured in terms of the virus titer difference between treated and untreated infected control eggs and inhibition percentage (IP). The EID50s of the virus stock suspension used in these series of experiments and extract/virus suspension related to both plants were 10-4.25/mL and 10-2.5/mL EID50, respectively. Finally, antiviral activity was determined as 10 1.75, which indicated the ability of the extracts to reduce the viral potency by more than 56folds. Both extracts were found effective.

**Table 1.** Measuring the Activity of *A. millefolium *Extracts by the Reed and Muench Method (100 μL of Virus Stock Solution Was Inoculated)

Virus Dilutions	Infected Embryos (HA Positive)		Non Infected Embryos (HA Negative)		Accumulated Values of Infected Embryos		Accumulated Values of Non Infected Embryos		Ratio Infected/Total		% Infected Embryos	
	Poscont.^a^	A.m.^ a^	Pos. cont.	A.m.	Pos. cont.	A.m.	Pos. cont	A.m.	Pos. cont	A.m.	Pos. Cont.	A.m.
**10^-1^**	3	3	0	0	8	3	0	0	8/8	3/3	100	100
**10^-2^**	2	0	1	3	5	0	1	3	5/6	0/3	83	0
**10^-3^**	2	0	1	3	3	0	2	6	3/5	0/6	60	0
**10^-4^**	1	0	2	3	1	0	4	9	1/5	0/9	20	0
**10^-5^**	0	0	3	3	0	0	7	12	0/7	0/12	0	0

^a^ Abbreviations: Pos. cont, Positive control; A.m, *Achillea millefolium*

**Table 2.** Measuring the Antiviral Activity of *T. vulgari *Extracts by the Reed and Muench Method (100 μL of Virus Stock Solution Was Inoculated)

Virus Dilutions	Infected Embryos (HA Positive)		Non Infected Embryos (HA Negative)		Accumulated Values of Infected Embryos		Accumulated Values of Non Infected Embryos		Ratio Infected/Total		% Infected Embryos	
	Pos cont.^a^	T.v.^a^	Pos. cont.	T.v.	Pos. cont.	T.v.	Pos. cont	T.v.	Pos. cont	T.v.	Pos. Cont.	T.v.
**10^-1^**	3	3	0	0	8	3	0	0	8/8	3/3	100	100
**10^-2^**	2	0	1	3	5	0	1	3	5/6	0/3	83	0
**10^-3^**	2	0	1	3	3	0	2	6	3/5	0/6	60	0
**10^-4^**	1	0	2	3	1	0	4	9	1/5	0/9	20	0
**10^-5^**	0	0	3	3	0	0	7	12	0/7	0/12	0	0

^a^ Abbreviations: Pos. cont; Positive control, T.v, *Thymus vulgaris*

## 5. Discussion

Nowadays natural products such as pure compounds and also plant extract scan provide unlimited opportunities for new antiviral drugs (13). Infectious viral diseases have remained important global issues for animals and humans. According to the dependency of viruses on host cells, only a few effective antiviral drugs are available to treat viral diseases. Nowadays finding new substances with intracellular and also extracellular antiviral activities is a need. These substances must affect viruses without harming the host cells. *In ovo* injection method was designed because studying the plants *in vitro* and *in vivo* is time consuming and expensive.. In this method, the plants were first screened for antiviral activity *in ovo* and then the best plants were selected for the next step. 

The amount of active constituents of the plants depend on the geographical distribution, season of collection, and climatic and ecological condition of the collection site (14). Studies show that many plants that are used in traditional medicine to treat viral diseases contain high levels of compounds such as alkaloids, terpenes, flavonoids, naphthoquinones, coumarins and anthraquinones (15). The mechanism of action of these compounds is killing the virus and/or interfering with viral replication. The most important glycoproteins in NDV are haemaglutinin neuramidase and fusion proteins, which are necessary for attachment and multiplication. Protease inhibition activity is observed in some substances of these plants, thus these compounds can interfere with the cleavage of these glycoproteins and inhibit virus attachment (15).

*T. vulgaris* (thyme) has essential oils which contain mixtures of different volatile and lipophilic substances, such as sesquiterpenes, monoterpenes, and phenylpropanoids. These substances have been proposed to be part of the preformed defense system of higher plants (13). Reports indicate that these substances can be active against microorganisms such as fungi, yeasts, viruses and bacteria. The present study revealed that extracts of the plants under study could be active against NDV and reduce virus yield. In another study, *T. vulgaris* was found effective against HSV-1/HSV-2. This plant, with the help of essential oils, interferes with the virus envelope, and also masks viral components which are necessary for attachment, penetration, or entry into host cells (13). 

Chemical analyses of *A. millefolium* show the presence of essential oils, tannins, flavonoids, sesquiterpene lactones, alkamides, inulin and ascorbic acid in the plant. The essential oil of *A. millefolium* possesses antioxidant and antimicrobial properties. This plant also reduces virus titer in *vivo*(16). The NDV is an enveloped virus and presumably these plants deactivate this virus by affecting the virus envelop. These plants could be used as additives in bird food to reduce the effectiveness of the virus; however, this should be proved *in vitro* and *in vivo*.
